# Down Regulation of a Gene for Cadherin, but Not Alkaline Phosphatase, Associated with Cry1Ab Resistance in the Sugarcane Borer *Diatraea saccharalis*

**DOI:** 10.1371/journal.pone.0025783

**Published:** 2011-10-03

**Authors:** Yunlong Yang, Yu Cheng Zhu, James Ottea, Claudia Husseneder, B. Rogers Leonard, Craig Abel, Randall Luttrell, Fangneng Huang

**Affiliations:** 1 Department of Entomology, Louisiana State University Agricultural Center, Baton Rouge, Louisiana, United States of America; 2 Southern Insect Management Research Unit, Agricultural Research Service, United States Department of Agriculture, Stoneville, Mississippi, United States of America; 3 Corn Insects and Crop Genetics Research Unit, Agricultural Research Service, United States Department of Agriculture, Ames, Iowa, United States of America; University of Kansas Medical Center, United States of America

## Abstract

The sugarcane borer, *Diatraea saccharalis,* is a major target pest of transgenic corn expressing *Bacillus thuringiensis* (Bt) proteins (i.e., Cry1Ab) in South America and the mid-southern region of the United States. Evolution of insecticide resistance in such target pests is a major threat to the durability of transgenic Bt crops. Understanding the pests' resistance mechanisms will facilitate development of effective strategies for delaying or countering resistance. Alterations in expression of cadherin- and alkaline phosphatase (ALP) have been associated with Bt resistance in several species of pest insects. In this study, neither the activity nor gene regulation of ALP was associated with Cry1Ab resistance in *D. saccharalis*. Total ALP enzymatic activity was similar between Cry1Ab-susceptible (Cry1Ab-SS) and -resistant (Cry1Ab-RR) strains of *D. saccharalis*. In addition, expression levels of three ALP genes were also similar between Cry1Ab-SS and -RR, and cDNA sequences did not differ between susceptible and resistant larvae. In contrast, altered expression of a midgut cadherin (DsCAD1) was associated with the Cry1Ab resistance. Whereas cDNA sequences of DsCAD1 were identical between the two strains, the transcript abundance of DsCAD1 was significantly lower in Cry1Ab-RR. To verify the involvement of DsCAD1 in susceptibility to Cry1Ab, RNA interference (RNAi) was employed to knock-down DsCAD1 expression in the susceptible larvae. Down-regulation of DsCAD1 expression by RNAi was functionally correlated with a decrease in Cry1Ab susceptibility. These results suggest that down-regulation of DsCAD1 is associated with resistance to Cry1Ab in *D. saccharalis*.

## Introduction

Evolution of insecticide resistance in target pests threatens the durability of transgenic crops expressing toxins from *Bacillus thuringiensis* (Bt). To date, field resistance that resulted in control failures or reduced efficacy of Bt crops has been documented in several target pest species of Bt corn and Bt cotton [1], [2], [3], [4]. Knowledge of Bt resistance mechanism is essential in understanding Bt resistance evolution and for developing effective management strategies. The most common mechanism of Bt resistance in the insect species that have been investigated is cadherin-mediated resistance. Recent studies [5], [6] showed that reduced level of membrane-bound ALPs is also associated with three major pests targeted by Bt crops including *Heliothis virescens*, *Helicoverpa armigera*, and *Spodoptera frugiperda* [5], [6].

The sugarcane borer, *Diatraea saccharalis*, is a major corn pest and a primary target of Bt corn in South America and the mid-southern region of the United States [7], [8], [9]. In these areas, >10 mha of Bt corn were planted for managing lepidopteran corn pests including *D. saccharalis* during 2010 [10], [11]. Molecular mechanisms of Bt resistance in stalk boring pests of corn (such as *D. saccharalis)* are poorly understood partly because highly resistant strains that could survive on commercial Cry1Ab corn hybrids were not available. Recently, a Cry1Ab-resistant (Cry1Ab-RR) strain of *D. saccharalis* has been established in the laboratory using a F_2_ screening method [12]. This Bt-resistant strain is able to complete larval development on commercial Cry1Ab corn plants, and is an ideal subject for studying Bt resistance mechanisms in corn stalk boring species.

Our previous study with this strain has shown that reduced expression of genes encoding three midgut aminopeptidases N (APNs) is associated with Cry1Ab resistance [13]. The current study is designed to test whether two other known mechanisms of Bt resistance (i.e., cadherin- and ALP-mediated resistance) are also associated with Cry1Ab resistance in *D. saccharalis*. Major objectives include analysis of total ALP activity, sequencing and examination of full-length cDNAs encoding three ALP and one cadherin proteins, and comparison of expression of genes encoding these proteins between Cry1Ab-susceptible (Cry1Ab-SS) and -RR strains of *D. saccharalis*. In addition, after establishing an association between reduced cadherin gene expression and Cry1Ab resistance, RNA interference (RNAi) was applied to knock-down cadherin gene expression in Cry1Ab-SS strain, and establish a functional correlation between susceptibility to Bt and reduced cadherin expression.

## Materials and Methods

### Insect strains

A Cry1Ab-resistant strain (Cry1Ab-RR) of *D. saccharalis* was established from a single two-parent family-line collected in a corn field near Winnsboro, Louisiana (Franklin Parish) during 2004 [12]. This resistant strain was shown to carry a major resistance allele identified using a F_2_ screen method. It has a significant resistance (∼100-fold) to purified trypsin-activated Cry1Ab toxin [14], and individuals of the Cry1Ab-RR strain can complete larval development on commercial Bt corn hybrids expressing the Cry1Ab protein. A Cry1Ab-susceptible strain (Cry1Ab-SS) of *D. saccharalis* was also developed from the same location. The Cry1Ab-RR strain had been backcrossed with the Cry1Ab-SS strain and re-selected on Cry1Ab corn leaf tissue for 3–4 times before it was used in this study.

### Quantification of alkaline phosphatase (ALP) activities

To examine the ALP activity, 3^rd^, 4^th^, or 5^th^ instars from both Cry1Ab-SS and -RR strains of *D. saccharalis* were dissected in cold 0.1 M Tris-HCl buffer (pH 8.0) to obtain entire guts and gut contents. Each gut was homogenized individually with 50 μl of 0.1 M Tris-HCl buffer (pH 8.0) and centrifuged at 10,000 *g* for 5 min at 4°C. The quantity of protein in homogenate supernatants was determined by the method of Bradford [15] with bovine serum albumin as the standard. Specific ALP enzymatic activities of midgut homogenates were measured with *p*-nitrophenyl phosphate disodium (*p*NPP) (Sigma) as substrate using the method described by Jurat-Fuentes and Adang [5]. Diluted proteins (1 mg/ml) were mixed with *p*NPP (1.25 mM, final concentration), and change in optical density (OD) at 405 nm was monitored for 15 min with reading interval of 15 s at 37°C in a microplate reader (ELx808iu; BioTek). Activity of ALP was defined as the amount of enzyme producing 1 µmol of chromogenic product *p*-nitrophenol (*p*-NP) min^−1^ mg of protein^−1^ at 37°C. The extinction coefficient of *p*-NP (17.8 mM^−1^ cm^−1^) was used for calculation of the specific ALP activities. For each combination of insect strain and instar, ALP activity was measured from ten reactions (replications) and two readings (sub-samples) for each reaction. Specific ALP activities are presented as means and standard errors of the mean (±SEM). Activity data were analyzed with a two-way analysis of variance (ANOVA) using the GLM procedure [16]. Treatment means were separated using LSMEANS tests at the α = 0.05 level [16].

### cDNA library construction

To clone full-length ALP and cadherin cDNAs, cDNA libraries of Cry1Ab-SS and -RR strains of *D. saccharalis* were constructed, sequenced, and subjected to Blast search of Genbank databases as described in [13]. Guts from ten 4^th^ instars of each of the Cry1Ab-SS and -RR strains of *D. saccharalis* were dissected and homogenized using TRIzol reagent (Invitrogen). Total RNA was precipitated from the homogenates according to the manufacturer's protocols. By using NucleoTrap mRNA purification kit (Clontech), mRNA was purified and used as template for the first strand cDNA synthesis. To ensure an appropriate yield of double strand cDNAs, these cDNAs were synthesized using a combination of primer extension and PCR amplification. For cDNA library construction, cDNAs were ligated into pDNR-LIB vector using Creator SMART cDNA Library Construction kit (Clontech). The ligation products were used to transform TOP10 competent cells (Invitrogen). Approximately 12,500 clones were obtained and sequenced using an ABI 3730XL DNA analyzer. Annotation of the transcriptomes was performed by using BlastX-NR similarity search of GenBank nucleotide database at National Center for Biotechnology Information (NCBI) [17].

### Cloning full-length cDNAs coding for three ALPs and one cadherin proteins of *D. saccharalis*

BlastX similarity searches of both the cDNA libraries yielded 30 and three clones that matched the ALP and cadherin genes in GenBank, respectively. By using SeqMan module of the Lasergene (DNAStar), these ALP and cadherin cDNA clones were assembled into cDNAs partially coding for three different ALPs and one cadherin, which were designated DsALP1, DsALP2, DsALP3, and DsCAD1, respectively. Cloning the full-length cDNAs of the DsALPs and DsCAD1 was achieved using the similar procedures as described in [18] with some modifications as described in [13]. Briefly, to obtain the full length cDNAs, total RNA was extracted from guts of 3^rd^ instars from Cry1Ab-SS and -RR strains as described above. The concentration of total RNA was determined using NanoDrop spectrophotometer (Thermo Fisher Scientific). The SuperScript First Strand cDNA Synthesis kit (Invitrogen) was used in a reverse transcriptase polymerase chain reaction (RT-PCR) with 5 µg of total RNA and an oligo-dT primer for cDNA synthesis. The template RNA was removed by adding 1 µl RNase H (2 U) after cDNA synthesis.

For cloning the three relatively short DsALPs cDNAs (∼1.5 kb), specific primers (Table S1) were directly designed to clone the major portions of cDNAs based on the corresponding sequences from the libraries. These primers were then used to directly amplify cDNA fragments with complete 3′-ends via PCR reactions. The 5′-end of the cDNA for each of the three DsALPs was obtained by using the 5′ rapid amplification of cDNA end (5′ RACE) system (Invitrogen). Based on the cDNA sequence obtained from cDNA libraries, two specific reverse primers for each of the three DsALPs (Table S1) were designed and used in the semi-nested amplifications with a forward abridged anchor primer from 5′-RACE kit. The 5′-end of the cDNA was isolated and C-tailed, and then was cloned into a pGEM-T vector. Plasmid DNAs were prepared and sequenced using an ABI 3730XL DNA analyzer to confirm the full coding sequences of the three DsALPs.

To determine the major cDNA sequence of the relatively long DsCAD1 (∼5 kb), four degenerate forward primers (Table S2) were designed based on the conserved regions of 10 cadherin cDNAs previously identified in other lepidopteran species. The amino acid sequences of the four conserved regions of the cadherin genes were ITQRQDYE, LINWNDE, ATDIDGP, and DEDGLHAG. In addition, one specific reverse primer, DsCAD1R0 (Table S2), was also designed based on the partial DsCAD1 sequence from the cDNA libraries. In each PCR reaction, a fragment of expected size was amplified using one degenerate forward and one degenerate/specific reverse primers. The 3′-end of the cDNA was amplified with a specific forward primers, named DsCAD1F2 (Table S2) along with the oligo-dT primer, while the 5′-end of the cDNA was obtained by using the 5′ RACE system with two specific reverse primers (Table S2) and sequenced following the same protocol described above.

To obtain error-proof full-length cDNAs for the three DsALPs and DsCAD1, a thermal-stable proof-reading Platinum High Fidelity Taq DNA polymerase (Invitrogen) was used in the subsequent PCR reactions. Total RNAs extracted from Cry1Ab-SS and -RR strains were used for synthesizing RT-cDNA. Three pairs of specific primers for the three DsALPs, flanking the 5′-and 3′-untranslated regions (Table S1), were used to re-amplify each of the three cDNAs. To obtain full length of DsCAD1 cDNA, three pairs of primers were designed (Table S2) with an average of 150 bp overlapping. The full-length cDNAs for each gene were re-amplified using the RT-cDNAs from Cry1Ab-SS and -RR strains, respectively. The PCR products were purified using Qiaquick PCR purification kit (Qiagen) and sequenced from both directions as described above.

### Identification and phylogenetic analyses of three DsALPs and DsCAD1

Presence of a signal peptide at the N-terminus of the deduced protein sequence of three DsALPs and DsCAD1 was determined using the SignalP 3.0 Server (http://www.cbs.dtu.dk/services/SignalP), while molecular weight and protein isoelectricpoints were predicted using the ExPASy Compute pI/Mw tool (http://ca.expasy.org/tools/pi_tool.html) [19]. Analysis of deduced protein sequences was conducted in the Myhits server (http://myhits.isbsib.ch/cgi-bin/motif_scan). Two GPI modification site prediction servers (PredGPI: http://gpcr2.biocomp.unibo.it/gpipe/pred.htm and GPI-SOM: http://gpi.unibe.ch/) were used to predict the GPI-anchor signal sequence and GPI anchoring sites of the three DsALPs. Presence of N- and O-glycosylation on the predicted protein sequences of three DsALPs and DsCAD1 was tested using the NetNGlyc 1.0 and NetOGlyc 3.1 servers (http://www.cbs.dtu.dk/services), respectively. Sequence-similarity analyses were performed using Blast through the NCBI [17]. Sequence comparisons were conducted using the ClustalW [20]. The Molecular Evolutionary Genetics Analysis (MEGA) (Ver. 4.1) [21] was used to perform multiple-sequence alignments and to examine phylogenetic relationships with the ALP or with the cadherin amino acid sequences of other lepidopteran species available in the GenBank.

### Quantitative real-time PCR

Total RNAs from 3^rd^ and 5^th^ instars of the Cry1Ab-SS and -RR strains of *D. saccharalis* were extracted as described above. Total RNAs then were treated with 2 μl DNaseI (1 mg/ml) (Boehringer Mannheim GmbH) at 37°C for 1 h to remove any residual DNA. For each treatment replication, three guts were pooled and total RNAs were extracted from the pooled samples. Concentrations of total RNAs, as measured with the NanoDrop spectrophotometer, were adjusted to 1 ng/µl. The iScript One-Step RT-PCR Kit with SYBR green (Bio-Rad) was used in a 25 µl reaction of Quantitative real-time PCR (qRT-PCR). For each combination of instar and insect strain, there were three replications in the qRT-PCR analysis. To obtain the absolute mRNA quantities of the three DsALPs and DsCAD1, two qRT-PCRs were performed for each gene as described in [13]. The ribosomal 18S gene was used to estimate RNA concentration for each sample. One pair of primers, Ds18SF1 and Ds18SR1 (Table S1), was designed based on the house-keeping 18S gene of *D. saccharalis*. Full-length cDNA of the 18S gene was prepared and used as internal standard in the first qRT-PCR. Serial dilutions (0.5, 5, 50, 500, and 5000 pg/µl) of the 18S internal standard and a negative control without the 18S cDNA were used to establish a standard curve. Five μl of total RNA template (1 ng/µl) or 18S internal standard were used in the qRT-PCR reaction. The threshold cycle (C_t_) value for each dilution was then plotted against the log of the cDNA quantity. The absolute quantities of cDNA equivalents of total RNAs were calculated from the standard curve. All initial total RNA samples were standardized and then used in the subsequent qRT-PCR.

For the second qRT-PCR, one specific primer pair for each of the three DsALPs (Table S1) and DsCAD1 (Table S2) was designed to produce amplicons of 139, 145, 143, and 112 bp, respectively. A partial cDNA fragment for each of these four genes was amplified and prepared as internal standards. Serial dilutions of internal standard (0.001, 0.01, 0.1, 1, and 10 pg/µl) were used to establish a standard curve as described above. Five microliters of the internal standard solution or standardized total RNA templates (10 ng/µl) were added into each reaction along with a negative control that contains all components and 5 µl ddH_2_O to replace the RNA template. Upon completion of the qRT-PCR, a dissociation curve analysis was conducted to verify the absence of any nonspecific amplicons. Based on the internal standard curve of the second qRT-PCR, absolute quantities of RT-cDNA of the three DsALPs and DsCAD1 were compared at 3^rd^ and 5^th^ instars between Cry1Ab-SS and -RR strains. There were 3–4 replications for each treatment. The transcript levels were presented as means and standard errors of the mean (±SEM). Results from gene expression assays were analyzed with a three-way (for DsALPs) or a two-way (for DsCAD1) ANOVA using the GLM procedure [16]. Treatment means were separated using LSMEANS tests at the α = 0.05 level [16].

### RNA interference of DsCAD1

To examine the functional linkage between the down-regulation of DsCAD1 and Cry1Ab resistance, RNA interference (RNAi) was used first to knock down DsCAD1 in the Cry1Ab-SS strain and then to assess susceptibility change using a bioassay. One pair of DsCAD1-specific primers, both containing the T7 promoter sequence (5′-TAATACGACTCACTATAGGG-3′), was designed to flank to position 488 to 888 of DsCAD1. DsCAD1 cDNA was used as template in PCR reaction to amplify a 401-bp fragment. The expected size of this PCR product was verified on an agarose gel and then used for *in vitro* transcription of the double-stranded RNA (dsRNA) using the MEGAScript RNAi kit (Ambion) based on the manufacturer's protocol. After being purified with the Minelute kit (Qiagen), the dsRNA was diluted in an elution solution (ES) (10 mM Tris-Cl with 1 mM EDTA, pH 7) and quantified using Nanodrop spectrophotometer as describe above.

To analyze gene expression of DsCAD1 after RNAi, oral delivery of dsRNA [22], [23] was applied as described in [13]. The non-invasive oral delivery of dsRNA has recently become a more attractive method in RNAi studies despite reports that it is less effective than dsRNA micro-injection [24], [25], [26]. To increase RNAi effectiveness and reduce variability in individual responses, a relatively high dose of dsRNA (250 ng) for each 3^rd^ instar was used for dsRNA feeding. Briefly, 3^rd^ instars from the Cry1Ab-SS strain were droplet-fed with 0.5 µl of the ES (control) or 0.5 µl ES containing 250 ng dsRNA (RNAi-treated). After 3 h, the droplet-fed larvae were transferred to individual cups containing 1 g of the meridic diet, and held in an environmental chamber maintained at 28°C, 50% RH, and a photoperiod of 16∶8 (L:D) h. At 24 h post feeding, guts were dissected to obtain total RNA, and DsCAD1 transcript levels of RNAi-treated and control larvae were determined by qRT-PCRs as described above.

Cry1Ab susceptibilities of RNAi-treated and control larvae of *D. saccharalis* were measured using a standard method of diet incorporating Bt toxin described in [14]. A Cry1Ab concentration of 16 µg g diet^−1^ was used for these tests. Treatment with this concentration was shown previously [14] to cause ∼50% and ∼8% mortality to larvae from the Cry1Ab-SS and -RR strains, respectively, in 7 days. To examine larval susceptibility to Cry1Ab toxin after RNAi, ES only, or ES containing 50 ng dsRNA was droplet-fed to neonates (<24 h old) of the Cry1Ab-SS strain. The droplet-fed neonates were placed in cells of 128-cell trays (C-D international) individually and rested for 3 h before they were used for bioassays. In the bioassays, approximately one gram of regular diet (non-treated control) or diet treated with purified trypsin-activated Cry1Ab toxin at the concentration of 16 µg g^−1^ diet was dispensed into each cell of the 128-cell trays (C–D International). One larva was then placed on the diet surface in each cell. The bioassay trays were held in an environmental chamber maintained as described above. Larval mortality was checked after 7 days. The 7-d mortality data were corrected based on the mortality observed on the control diet [27]. There were three replications for each treatment with 48 larvae in each replicate. The corrected mortalities were presented as means and standard errors of the mean (±SEM). A one-way ANOVA was performed to determine treatment differences at α = 0.05 level [16].

## Results

### Enzymatic and molecular comparisons of three DsALP proteins, cDNAs and gene transcripts

**Figure 1:**
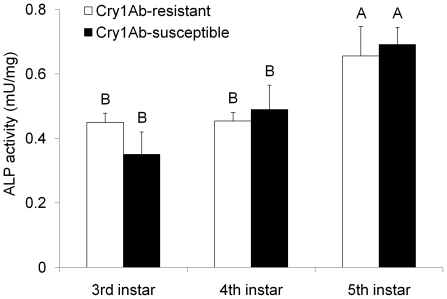
Total alkaline phosphatase (ALP) activity of whole midgut (gut tissue plus gut contents in the lumen) in different instars of Cry1Ab-susceptible and -resistant strains of *D. saccharalis*. Bars represent the means and standard errors of ten gut samples from a total of eight different larvae. Mean values in the figure followed by a different letter are significantly different (*P*<0.05, LSMEANS tests).

Activities of ALPs from Cry1Ab-SS and -RR strains of *D. saccharalis* were not significantly different at each instar (F = 0.01; df = 1, 44; *P* = 0.9279) (Fig. 1). The interaction effect of insect strain and instar was also not significant (F = 0.91; df = 2, 44; *P* = 0.4104). Total ALP activity increased as *D. saccharalis* larvae aged from 3^rd^ to 5^th^ instars (F = 11.14; df = 2, 44; *P* = 0.0001) (Fig. 1), and the increase in 5^th^ instars was significant (*P*<0.05) compared to that measured from 3^rd^ and 4^th^ instars.

**Figure 2:**
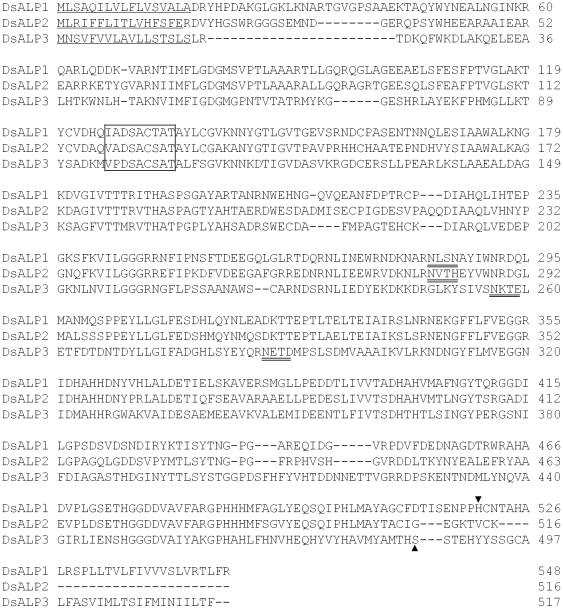
Deduced amino acid sequences of three alkaline phosphatase isoforms (DsALP1, DsALP2, and DsALP3) in *D. saccharalis* aligned by ClustalW. The predicted signal peptide sequences are single-underlined. Potential N-glycosylation sites are double-underlined. Alkaline phosphatase active domains are indicated inside the rectangle. Predicted GPI anchor sites are indicated by filled-black triangles.

Similarly, no differences in cDNA sequences of three DsALPs were detected between Cry1Ab-SS and -RR strains of *D. saccharalis* (Fig. 2). Pairwise alignments showed that cDNA sequences of the three DsALPs in the Cry1Ab-SS and -RR strains of *D. saccharalis* were identical. No deletions or insertions and no base substitutions were detected in the cDNAs of the three DsALPs between the two strains.

The similarity in amino acid sequence among the three DsALPs was low (∼28.8%) among each other, indicating that they may represent different variants. The open reading frames (ORFs) of the three DsALPs were 1647 (DsALP1), 1551(DsALP1), and 1554 bp (DsALP1), encoding putative proteins of 548, 516, and 517 amino acid residues, respectively. The predicted molecular weights were 60.4, 57.0, and 57.2 kDa for DsALP1, DsALP2, and DsALP3, respectively. The isoelectric points of the three DsALPs were 5.59, 5.24, and 5.55, respectively. Hydrophobic signal sequences contained 17, 16, and 17 amino acids, respectively and they were found in the N-terminal region of the three DsALPs (Fig. 2), which is consistent with previous reports of ALPs from other insect species. The GPI-anchor signal sequences observed in the ALPs of most other insect species were detected in the C-terminal regions of DsALP1 and DsALP3, but not DsALP2 (Fig. 2), suggesting that DsALP1 and DsALP3 are membrane-bound ALPs whereas DsALP2 is a soluble ALP. All three DsALPs contained a predicted phosphatase domain (I(V)A(P)DS*ACT(S)AT) with S* being the enzymatic active site in the highly conserved protein sequence regions. One potential N-glycosylation site was found in each of the three DsALP proteins (^283^NLSN^286^ and ^280^NVTH^283^, and ^256^NKTE^259^, respectively). Another potential N-glycosylation site was also observed at ^287^NETD^290^ in DsALP3 (Fig. 2). No potential O-glycosylation sites were detected in any of the three DsALPs from *D. saccharalis*.

**Figure 3:**
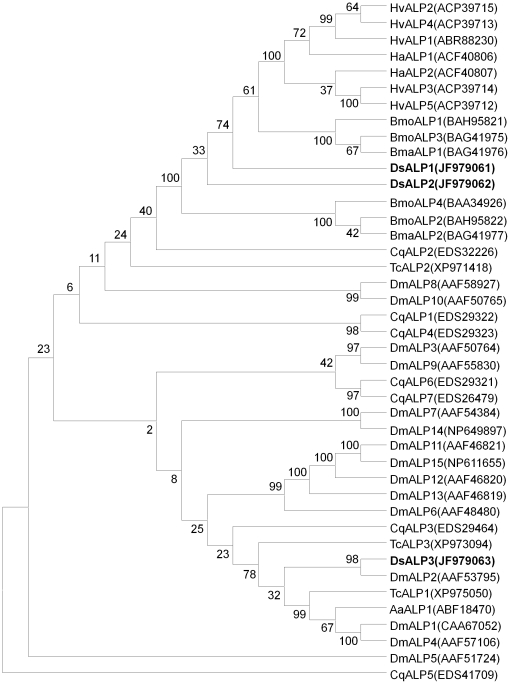
A phylogenetic tree generated by ClustalW alignment of alkaline phosphatase amino acid sequences from insect species using MEGA. The bootstrap values expressed as percentages of 500 replications, are shown at branch points. GenBank accession numbers are displayed within the tree. Abbreviations: Aa, *Aedes aegypti*; Bma, *Bombyx mandarina*; Bmo, *Bombyx mori*; Cq, *Culex quinquefasciatus*; Dm, *Drosophila melanogaster*; Ds, *Diatraea saccharalis*; Ha, *Helicoverpa armigera*; Hv, *Heliothis virescens*; Tc, *Tribolium castaneum*.

In a phylogenetic tree constructed based on alignments of 39 ALPs from eight other insect species, the three DsALPs from *D. saccharalis* were located in three separated groups and all shared a low sequence identity (ranged from 34.1% to 57.5%) (Fig. 3). Amino acid sequences of DsALP1 and DsALP2 were highly similar to membrane-bound ALPs from four lepidopteran species, *Bombyx mori*, *B. mandarina*, *H. virescens*, and *H. armigera* (Fig. 3), whereas DsALP3 was less similar to the ALPs from other insect species but had high homology with DmALP2 from *Drosophila melanogaster*.

**Figure 4:**
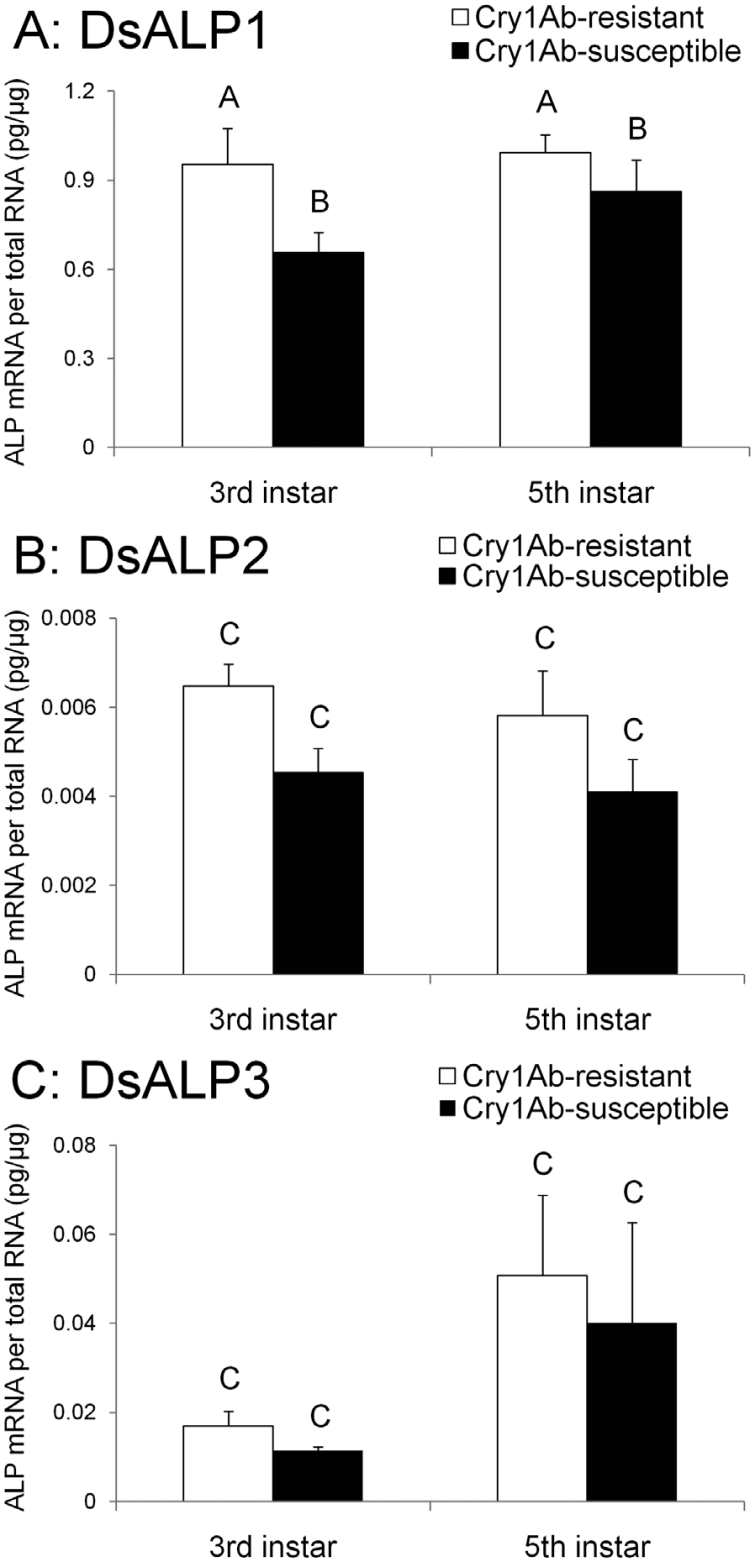
Gene expression levels of three alkaline phosphatase (ALP) genes (A: DsALP1, B: DsALP2, and C: DsALP3) of 3^rd^ and 5^th^ instars of Cry1Ab-susceptible and -resistant strains of *D. saccharalis*. Absolute transcript abundance (ALP mRNA per total RNA (pg/μg)) was determined using qRT-PCR with SYBR. Bars represent the means and standard errors of 4 total RNA samples each containing a pool of total RNAs from three larvae. Mean values in the three figures followed by a different letter are significantly different (*P*<0.05, LSMEANS tests).

Levels of transcription were significantly higher for the DsALP1 gene than DsALP2 or DsALP3 (F = 373.70; df = 2, 42; *P*<0.0001) (Fig. 4). Whereas Cry1Ab-RR had a significantly higher level of DsALP1 transcript than Cry1Ab-SS (Fig. 4), there were no significant differences between strains in DsALP2 and DsALP3 gene expression.

### Molecular characterization and functional analysis of DsCAD1 gene

#### cDNA sequences of DsCAD1 were identical between Cry1Ab-SS and -RR strains of *D. saccharalis*

**Figure 5:**
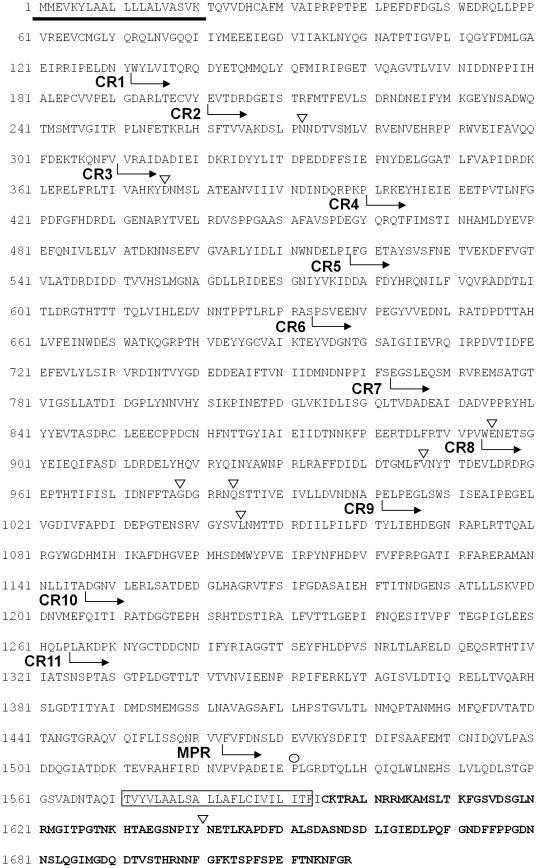
Deduced amino acid sequence of a cadherin-like DsCAD1 gene from *D. saccharalis*. The putative signal peptide sequences are underlined and the transmembrane (TM) spanning regions are boxed. Filled-black triangles denote the predicted putative N-glycosylation sites. Open triangle denotes the putative amidation site and open circle represents the leucine zipper. Also shown are cadherin repeats (CR1-CR11) and the membrane-proximal region (MPR). The bold sequence at the C-terminal sequence represents the intracellular domain.

Pairwise alignment showed that cDNA sequences of DsCAD1 in the Cry1Ab-SS and -RR strains of *D. saccharalis* were identical. As observed for the three DsALPs, no deletions or insertions and no base substitutions were detected in the cDNAs of DsCAD1 between the two strains. A full-length cDNA of 5304 bp of DsCAD1 was cloned from both strains, which had an ORF of 5157 bp encoding a 1718 amino acid putative cadherin-like glycoprotein. At the 3′-end of the cDNA sequence, two poly-A signal sequences (AATAAA) were apparent at positions 15 bp and 169 bp upstream of the poly-A tail, respectively. The putative isoelectric point for the cadherin protein was 4.38 and the estimated molecular weight was 193 kDa. Sequence analysis showed that DsCAD1 contained a transmembrane region of 23 amino acids (Fig. 5). The extracellular domain comprised a signal sequence of 21 amino acid residues, 11 cadherin repeats (CR1-CR11), and a membrane-proximal region (Fig. 5). Seven putative N-glycosylation sites, an amidation site, and a leucine zipper motif were identified in the DsCAD1 protein sequence. The protein's intracellular domain was composed of 124 amino acid residues (Fig. 5). All the above sequences/domains matched the structural characteristics of cadherin genes from other lepidopterans that have been investigated [28], [29], [30].

**Figure 6:**
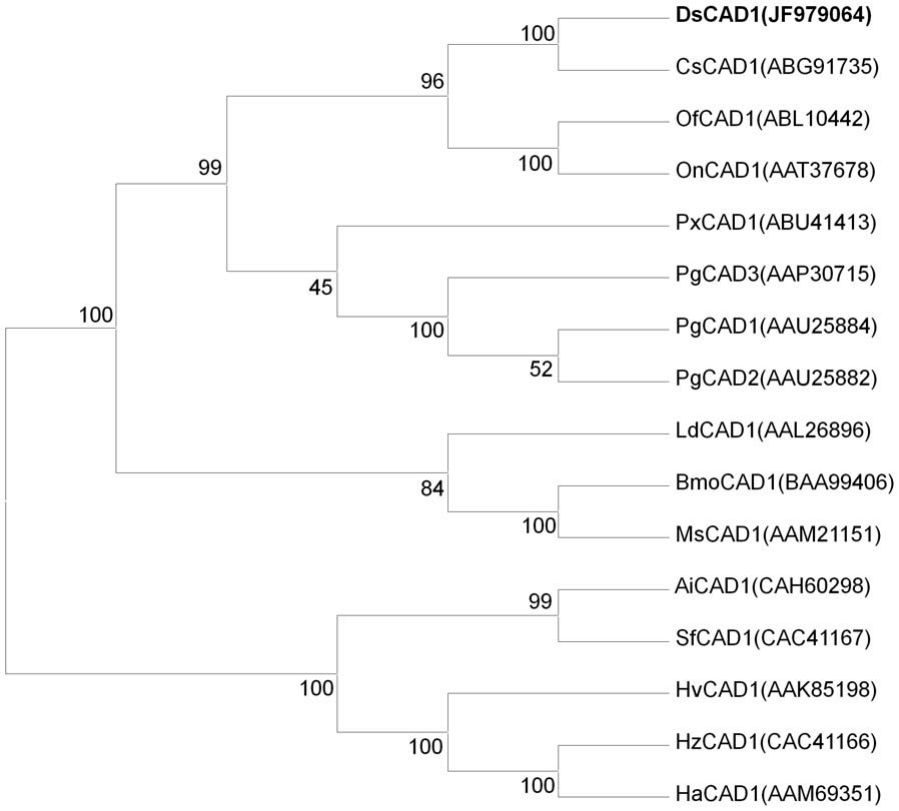
A phylogenetic tree generated by ClustalW alignment of cadherin amino acid sequences from lepidopteran species using MEGA. The bootstrap values expressed as percentages of 500 replications, are shown at branch points. GenBank accession numbers are displayed within the tree. Abbreviations: Ai, *Agrotis ipsilon*; Bm, *Bombyx mori*; Cs, *Chilo suppressalis*; Ds, *Diatraea saccharalis*; Ha, *Helicoverpa armigera*; Hv, *Heliothis virescens*; Hz, *Helicoverpa zea*; Ld, *Lymantria dispar*; Ms, *Manduca sexta*; Of, *Ostrinia funarcalis*; On, *Ostrinia nubilalis*; Pg, *Pectinophora gossypiella*; Px, *Plutella xylostella*; Sf, *Spodoptera frugiperda*.

A phylogenetic tree was generated based on alignment with 15 cadherins (Fig. 6) identified from 13 lepidopteran species in the GenBank. The phylogenetic analysis indicated that DsCAD1 in *D. saccharalis* is most closely related to six cadherins from *Chilo suppressalis* (Family Pyralidae) (ABG91735), *Ostrinia funarcalis* (Family Crambidae) (ABL10442), *Ostrinia nubilalis* (Family Crambidae) (AAT37678), and *Pectinophora gossypiella* (Family Gelechiidae) (AAU25884, AAU25882, and AAP30715), whereas DsCAD1 is more distantly related to cadherin genes identified from the remaining eight species (Fig. 6).

#### Expression of DsCAD1 is reduced in the Cry1Ab-RR strain

**Figure 7:**
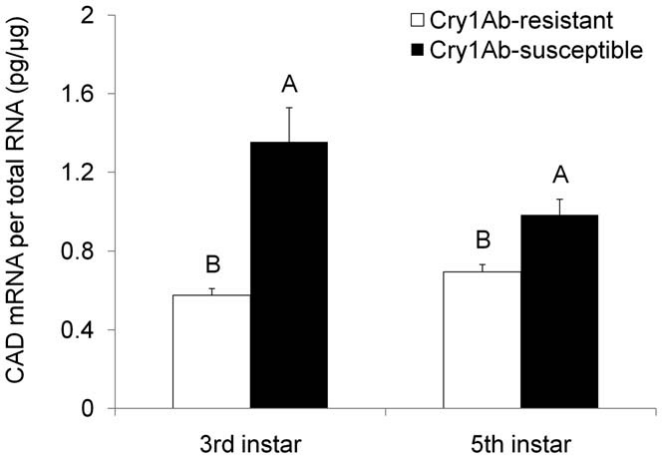
Gene expression levels of DsCAD1 in 3^rd^ and 5^th^ instars of the Cry1Ab-susceptible and -resistant strains of *D. saccharalis*. Absolute transcript abundance (cadherin mRNA per total RNA (pg/μg)) was determined by qRT-PCR using SYBR. Bars represent the means and standard errors of three total RNA samples. Each sample contains a pool of total RNAs from three larvae. Mean values in the figure followed by a different letter are significantly different (*P*<0.05, LSMEANS tests).

Expression of the DsCAD1 gene in the Cry1Ab-RR strain of *D. saccharalis* was significantly down-regulated compared to larvae of the same age from the Cry1Ab-SS strain for both 3^rd^ and 5^th^ instars (F = 19.40; df = 1,5; *P* = 0.0070) (Fig. 7). Compared to that in the Cry1Ab-SS strain, DsCAD1 mRNA in the Cry1Ab-RR strain was reduced by 57.6% for 3^rd^ instars and 29.3% for 5^th^ instars. The main effects of instar and the interaction of insect strain and instar on the gene expression levels were not significant (F = 1.69; df = 1,5; *P* = 0.2489 for instar and F = 5.46; df = 1,5; *P* = 0.0666 for the interaction of insect strain and instar).

#### Knocking-down expression of DsCAD1 with RNAi changes Cry1Ab susceptibility

**Figure 8:**
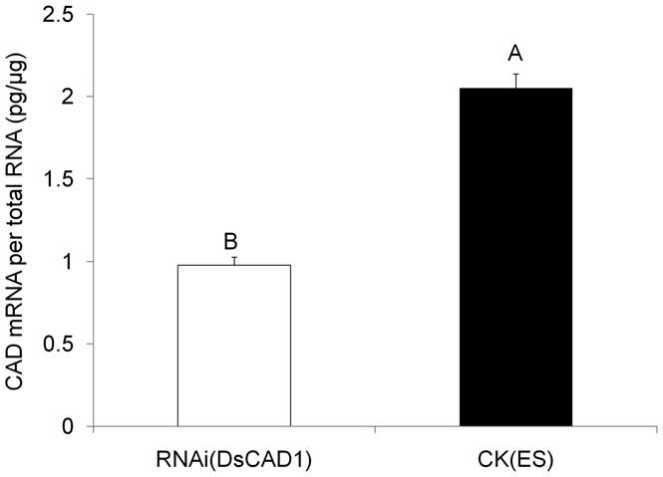
Gene expression levels of DsCAD1 after dsRNA feeding to 3^rd^ instars of the Cry1Ab-susceptible strain of *D. saccharalis*. The RNAi-treated 3^rd^ instars were droplet-fed elution solution (ES) containing 250 ng dsRNA for DsCAD1, while the control neonates (CK) were fed ES only. Absolute transcript abundance (cadherin mRNA per total RNA (pg/μg)) was determined by qRT-PCR using SYBR. Bars represent the means and standard errors of three total RNA samples. Each sample contains a pool of total RNAs from three larvae. Mean values followed by a different letter are significantly different (*P*<0.05, LSD tests).

**Figure 9:**
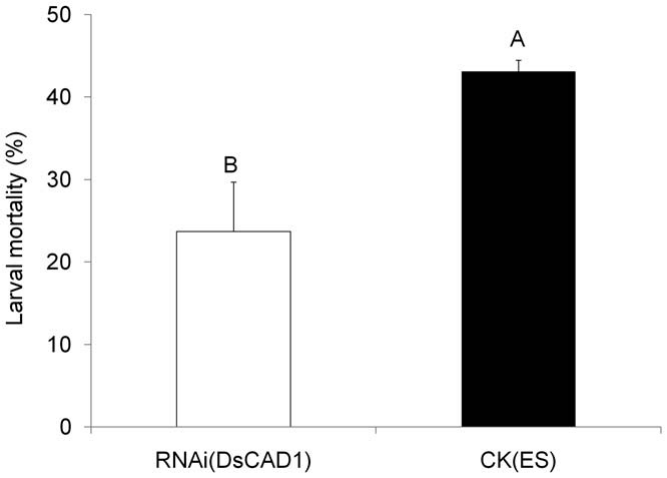
Larval mortality of the Cry1Ab-susceptible strain of *D. saccharalis* after RNAi for DsCAD1. The RNAi-treated neonates were droplet-fed 0.1 μl elution solution (ES) containing 50 ng cadherin dsRNA, while the control neonates (CK) were fed ES only before released for bioassays. The bioassays were conducted in three independent replications and each replicate for a treatment contained 48 neonates. Bars shown are the means of three experiments with their standard errors. Mean values followed by a different letter are significantly different (*P*<0.05, LSD tests).

Knock-down of the DsCAD1 gene by RNAi was observed in 3^rd^ instars of *D. saccharalis*. Analysis by qRT-PCR showed that the transcription of DsCAD1 in 3^rd^ instars from the Cry1Ab-SS strain was reduced by 52.2% at 24 h after treatment of larvae with 250 ng dsRNA (Fig. 8). This difference was statistically significant (F = 118.57; df = 1,4; *P* = 0.0004). In addition, mortality of RNAi-treated Cry1Ab-SS neonates fed diet containing 16 μg Cry1Ab g^-1^ diet was reduced significantly relative to that of ES-treated control larvae (F = 9.97; df = 1, 4; *P* = 0.0343) (Fig. 9). Mortality of ES-treated (control) larvae was 43.1% after feeding upon Cry1Ab- treated diet but only 23.7% for the RNAi-treated insects (Fig. 9).

## Discussion

The ALPs (EC 3.1.3.1) are mainly localized in microvilli of columnar cells and the midgut epithelium cells of insects [31], [32]. Insect ALPs have been proposed to function in active absorption of metabolites and transport processes as well as to participate in cell adhesion and differentiation in some cases [32], [33]. The ALPs can be grouped into those that are soluble or membrane-bound [32], [34], [35], and members from both groups are found in larval midgut epithelium cells. The two ALP groups are believed to have different functions *in vivo* due to the differences in enzymatic activity and the structure of the sugar side chain [36]. Membrane-bound ALPs are thought to be involved with digestion and absorption of nutrients, whereas soluble-ALPs may play a role in the regulation of ionic balance [32], [35]. Based on the lepidopteran ALP sequences available in GenBank, seven out of the 13 ALPs identified in four other lepidopteran species have a predicted GPI-anchoring site in their deduced protein sequences. Among those, all seven ALPs with a GPI-anchoring site were membrane-bound [35], [37], [38]. The GPI sequence signatures of DsALP1 and DsALP3 identified in *D. saccharalis* suggest that these two DsALPs may likely be membrane-bound, whereas the DsALP2 (without the possible GPI-anchoring site) is likely a soluble form. However, the phylogenetic analysis revealed that DsALP2 was grouped with GPI-anchored ALPs that were considered as Bt toxin binding receptors for *B. mori*, *B. mandarina*, *H. virescens*, and *H. armigera* [35], [37], [38].

In a previous study, interactions between Cry1Ac toxin and ALPs resulted in a decreased ALP enzymatic activity in *Manduca sexta* [39]. Several studies have demonstrated that membrane-bound ALPs in several lepidopteran species can act as Cry toxins binding proteins [5], [37], [40], [41]. In addition, recent studies have shown that reduced ALP gene expression is associated with Bt resistance in three major pests targeted by Bt crops [5], [6]. Results of this study did not show any reduction in expression of ALP activity in the Cry1Ab-RR strain of *D. saccharalis* compared to the Cry1Ab-SS larvae, although there was a trend toward increased transcription of the three DsALPs in the Cry1Ab-RR strain relative to the Cry1Ab-SS strain for both instars tested. Further, cDNA sequences of the three DsALP genes were identical between the Cry1Ab-SS and -RR strains. These data, together with similar ALP enzymatic activity data, suggest that the Cry1Ab resistance of *D. saccharalis* is not associated with mutations or reduced expression in ALP genes. It is possible that use of whole gut tissue in this study, instead of brush border membrane vesicles [6], might obscure the down-regulation of the ALPs. However, the successful detection of down-regulation of both DsCAD1 from this study and APNs [13] in the same tissue indirectly validated our ALP data.

Results of this study suggest that the Cry1Ab resistance in *D. saccharalis* is likely associated with the reduction in gene expression of DsCAD1. The cadherin-like protein identified from *D. saccharalis* demonstrated a relatively high similarity to other members of the cadherin super-family in lepidopteran species, indicating that the cadherin-like protein from *D. saccharalis* may share structures, functions, and consequently specificity for Cry1A toxins with other insects. Several previous reports showed that Cry1A resistance in several other lepidopteran species was associated with mutations of the cadherin genes resulting in either deletions (e.g., in *H. armigera* and *O. nubilalis*) [42], [43], [44] or premature stop codons (e.g., in *H. virescens*, *P. gossypiella*, and *O. nubilalis*) [44], [45], [46]. In addition, single amino acid mutations in the toxin-binding region of a cadherin protein in *H. virescens* caused a substantial decrease in toxin binding [47]. These results indicate that such single amino acid mutations or deletions can lead to high levels of Bt resistance in lepidopteran species. However, in the current study, we found no differences in the sequence of the cadherin cDNAs between the Cry1Ab-SS and -RR strains of *D. saccharalis*.

Numerous studies have suggested that APNs are receptors of Bt Cry toxins and are involved in Bt resistance in many insect species [48], [49], [50], [51], [52]. As observed for the cadherin gene, our previous study [13] also showed that cDNAs of three APN genes were identical between the Cry1Ab-SS and -RR strains of *D. saccharalis*, but the expression levels of all the three APN genes were significantly reduced in the resistant strain compared to those of the susceptible strain. Taken together, these results suggest that the reduction in expression of both the cadherin and APNs is associated with the Cry1Ab resistance in *D. saccharalis*. However, our finding does not exclude involvement of other genes in the resistance. Recently, we conducted a microarray analysis of 7,145 genes, which revealed 273 significantly up-regulated and 111 significantly down-regulated genes in Cry1Ab-RR strain. Our microarray analysis provided some interesting data, especially the up-regulation of large portion of metabolic or catalytic activity related genes in the Bt resistant strain (manuscript is in preparation). Future studies will focus on comparative characterizations of those differentially expressed genes and establish a linkage between gene regulation and Cry1Ab resistance in *D. saccharalis*.

## Supporting Information

Table S1 **Sequences of primers used in cDNA cloning and quantitative reverse transcriptase polymerase chain reaction (qRT-PCR) for characterization of three midgut alkaline phosphatase genes from Cry1Ab-susceptible and -resistant strains of** ***D. saccharalis*** **.**
(DOC)

Table S2 **Sequences of primers used in cDNA cloning, quantitative reverse transcriptase polymerase chain reaction (qRT-PCR), and double-stranded RNA (dsRNA) synthesis for characterization of a midgut cadherin gene from Cry1Ab-susceptible and -resistant strains of** ***D. saccharalis*** **.**
(DOC)

## References

[1] van Rensburg JBJ (2007). First report of field resistance by the stem borer, *Busseola fusca* (Fuller) to Bt-resistance maize.. S Afr J Plant Soil.

[2] Storer NP, Babcock JM, Schlenz M, Meade T, Thompson GD (2010). Discovery and characterization of field resistance to Bt maize: *Spodoptera frugiperda* (Lepidoptera: Noctuidae) in Puerto Rico.. J Econ Entomol.

[3] Dhurua S, Gujar GT (2011). Field-evolved resistance to Bt toxin Cry1Ac in the pink bollworm, *Pectinophora gossypiella* (Saunders) (Lepidoptera: Gelechiidae) from India.. http://dx.doi.org/10.1002/ps.2127.

[4] Tabashnik BE, Van Rensburg JB, Carrière Y (2009). Field-evolved insect resistance to Bt crops: definition, theory, and data.. J Econ Entomol.

[5] Jurat-Fuentes JL, Adang MJ (2004). Characterization of a Cry1Ac-receptor alkaline phosphatase in susceptible and resistant *Heliothis virescens* larvae.. Eur J Biochem.

[6] Jurat-Fuentes JL, Karumbaiah L, Jakka SRK, Ning C, Liu C (2011). Reduced levels of membrane-bound alkaline phosphatase are common to lepidopteran strains resistant to Cry toxins from *Bacillus thuringiensis*.. PLoS ONE.

[7] Huang F, Andow DA, Buschman LL (2011). Success of the high dose/refuge resistance management strategy after fifteen years of Bt crop use in North America.. Entomol Exp Appl.

[8] Castro BA, Riley TJ, Leonard BR, Baldwin J (2004). Borers galore: emerging pest in Louisiana corn, grain sorghum and rice.. LA Agric.

[9] Huang F, Leonard BR, Gable RH (2006). Comparative susceptibility of European corn borer, southwestern corn borer, and sugarcane borer (Lepidoptera: Crambidae) to Cry1Ab protein in a commercial Bt-corn hybrid.. J Econ Entomol.

[10] James C (2010). Global status of commercialized biotech/GM crops: 2010..

[11] National Agricultural Statistics Service (NASS) (2010). Acreage.. http://usda.mannlib.cornell.edu/usda/current/Acre/Acre-06-30-2010.pdf.

[12] Huang F, Leonard BR, Andow DA (2007a). Sugarcane borer (Lepidoptera: Crambidae) resistance to transgenic *Bacillus thuringiensis* maize.. J Econ Entomol.

[13] Yang Y, Zhu YC, Ottea J, Husseneder C, Leonard BR (2010). Molecular characterization and RNA interference of three midgut aminopeptidase N isozymes from *Bacillus thuringiensis*-susceptible and -resistant strains of sugarcane borer, *Diatraea saccharalis*.. Insect Biochem Mol Biol.

[14] Huang F, Leonard BR, Wu X (2007). Resistance of sugarcane borer to *Bacillus thuringiensis* Cry1Ab toxin.. Entomol Exp Appl.

[15] Bradford MM (1976). A rapid and sensitive method for the quantitation of microgram quantities of protein utilizing the principle of protein-dye binding.. Anal Biochem.

[16] SAS Institute (2008). SAS/STAT 9.2..

[17] Altschul SF, Madden TL, Schäffer AA, Zhang J, Zhang Z (1997). Gapped BLAST and PSI-BLAST: a new generation of protein database search programs.. Nucleic Acids Res.

[18] Zhu YC, Snodgrass GL, Chen MS (2004). Enhanced esterase gene expression and activity in a malathion-resistant strain of the tarnished plant bug, *Lygus lineolaris*.. Insect Biochem Mol Biol.

[19] Bendtsen JD, Nielsen H, von Heijne G, Brunak S (2004). Improved prediction of signal peptides: SignalP 3.0.. J Mol Biol.

[20] Thompson JD, Higgins DG, Gibson TJ (1994). CLUSTAL W: improving the sensitivity of progressive multiple sequence alignment through sequence weighting, positions-specific gap penalties and weight matrix choice.. Nucl Acids Res.

[21] Tamura K, Dudley J, Nei M, Kumar S (2007). MEGA4: Molecular Evolutionary Genetics Analysis (MEGA) software version 4.0.. Mol Biol Evol.

[22] Turner CT, Davy MW, MacDiarmid RM, Plummer KM, Birch NP (2006). RNA interference in the light brown apple moth, *Epiphyas postvittana* (Walker) induced by double-stranded RNA feeding.. Insect Mol Biol.

[23] Bautista MAM, Tadashi M, Miura K, Tanaka T (2009). RNA interference-mediated knockdown of a cytochrome P450, *CYP6BG1*, from the diamondback moth, *Plutella xylostella*, reduces larval resistance to permethrin.. Insect Biochem Mol Biol.

[24] Rajagopal R, Sivakumar S, Agrawal N, Malhotra PM, Bhatnagar RK (2002). Silencing of midgut aminopeptidase N of *Spodoptera litura* by double stranded RNA establishes its role as *Bacillus thuringiensis* receptor.. J Biol Chem.

[25] Araujo RN, Santos A, Pinto FS, Gontijo NF, Lehane MJ (2006). RNA interference of the salivary gland nitrophorin 2 in the triatomine bug *Rhodnius prolixus* (Hemiptera: Reduviidae) by dsRNA ingestion or injection.. Insect Biochem Mol Biol.

[26] Baum JA, Bogaert T, Clinton W, Heck GR, Feldmann P (2007). Control of coleopteran insect pests through RNA interference.. Nat Biotechnol.

[27] Abbott WS (1925). A method for computing the effectiveness of an insecticide.. J Econ Entomol.

[28] Nagamatsu Y, Koike T, Sasaki K, Yoshimoto A, Furukawa Y (1999). The cadherin-like protein is essential to specificity determination and cytotoxic action of the *Bacillus thuringiensis* insecticidal CryIAa toxin.. FEBS Lett.

[29] Hua G, Jurat-Fuentes JL, Adang MJ (2004). Bt-R1a extracellular cadherin repeat 12 mediates *Bacillus thuringiensis* Cry1Ab binding and cytotoxicity.. J Biol Chem.

[30] Flannagan RD, Yu CG, Mathis JP, Meyer TE, Shi X (2005). Identification, cloning and expression of a Cry1Ab cadherin receptor from European corn borer, *Ostrinia nubilalis* (Hübner) (Lepidoptera: Crambidae).. Insect Biochem Mol Biol.

[31] Wolfersberger MG (1984). Enzymology of plasma membranes of insect intestinal cells.. Am Zool.

[32] Eguchi M (1995). Alkaline phosphatase isozymes in insects and comparison with mammalian enzyme.. Comp Biochem Physiol.

[33] Chang W, Zachow K, Bentley D (1993). Expression of epithelial alkaline phosphatase in segmentally iterated bands during grasshopper limb morphogenesis.. Development.

[34] Itoh M, Takeda S, Yamamoto H, Izumi S, Tomino S (1991). Cloning and sequencing analysis of membrane-bound alkaline phosphatase cDNA of the silkworm, *Bombyx mori*.. Biochim Biophys Acta.

[35] Itoh M, Kanamori Y, Takao M, Eguchi M (1999). Cloning of soluble alkaline phosphatase cDNA and molecular basis of the polymorphic nature in alkaline phosphatase isozymes of *Bombyx mori* midgut.. Insect Biochem Mol Biol.

[36] Okada N, Azuma M, Eguchi M (1989). Alkaline phosphatase isozymes in the midgut of silkworm: purification of high pH-stable microvillus and labile cytosolic enzymes.. J Comp Physiol B.

[37] Ning C, Wu K, Liu C, Gao Y, Jurat-Fuentes JL (2010). Characterization of a Cry1Ac toxin-binding alkaline phosphatase in the midgut from *Helicoverpa armigera* (Hübner) larvae.. J Insect Physiol.

[38] Perera OP, Willis JD, Adang MJ, Jurat-Fuentes JL (2009). Cloning and characterization of the Cry1Ac-binding alkaline phosphatase (HvALP) from *Heliothis virescens*.. Insect Biochem Mol Biol.

[39] Sangadala S, Walters FS, English LH, Adang MJ (1994). A mixture of *Manduca sexta* aminopeptidase and phosphatase enhances *Bacillus thuringiensis* insecticidal CryIA(c) toxin binding and ^86^Rb^+^-K^+^ efflux in vitro.. J Biol Chem.

[40] Arenas I, Bravo A, Soberón M, Gómez I (2010). Role of alkaline phosphatase from *Manduca sexta* in the mechanism of action of *Bacillus thuringiensis* Cry1Ab toxin.. J Biol Chem.

[41] McNall RJ, Adang MJ (2003). Identification of novel *Bacillus thuringiensis* Cry1Ac binding proteins in *Manduca sexta* midgut through proteomic analysis.. Insect Biochem Mol Biol.

[42] Xu X, Yu L, Wu Y (2005). Disruption of a cadherin gene associated with resistance to Cry1Ac δ-endotoxin of *Bacillus thuringiensis* in *Helicoverpa armiger*a.. Appl Environ Microbiol.

[43] Yang Y, Chen H, Wu Y, Yang Y, Wu S (2007). Mutated cadherin alleles from a field population of *Helicoverpa armigera* confer resistance to *Bacillus thuringiensis* toxin Cry1Ac.. Appl Environ Microbiol.

[44] Bel Y, Siqueira HAA, Siegfried BD, Ferré J, Escriche B (2009). Variability in the cadherin gene in an *Ostrinia nubilalis* strain selected for Cry1Ab resistance.. Insect Biochem Mol Biol.

[45] Gahan LJ, Gould F, Heckel DG (2001). Identification of a gene associated with Bt resistance in *Heliothis virescens*.. Science.

[46] Morin S, Biggs RW, Sisterson MS, Shriver L, Ellers-Kirk C (2003). Three cadherin alleles associated with resistance to *Bacillus thuringiensis* in pink bollworm.. Proc Natl Acad Sci USA.

[47] Xie R, Zhuang M, Ross LS, Gómez I, Oltean DI (2005). Single amino acid mutations in the cadherin receptor from *Heliothis virescens* affect its toxin binding ability to Cry1A toxins.. J Biol Chem.

[48] Knight PJK, Crickmore N, Ellar DJ (1994). The receptor for *Bacillus thuringiensis* Cry1A(c) delta-endotoxin in the brush border membrane of the lepidopteran *Manduca sexta* is aminopeptidase N. Microbiol.

[49] Denolf P, Hendrickx K, Van Damme J, Jansens S, Peferoen M (1997). Cloning and characterization of *Manduca sexta* and *Plutella xylostella* midgut aminopeptidase N enzymes related to *Bacillus thuringiensis* toxin-binding proteins.. Eur J Biochem.

[50] Oltean DI, Pullikuth AK, Lee HK, Gill SS (1999). Partial purification and characterization of *Bacillus thuringiensis* Cry1A toxin receptor A from *Heliothis virescens* and cloning of the corresponding cDNA. Appl.. Environ Microbiol.

[51] Yaoi K, Nakanishi K, Kadotani T, Imamura M, Koizumi N (1999). cDNA cloning and expression of *Bacillus thuringiensis* Cry1Aa toxin binding 120 kDa aminopeptidase N from *Bombyx mori*.. Biochim Biophys Acta.

[52] Bravo A, Gomez I, Conde J, Munoz-Garay C, Sanchez J (2004). Oligomerization triggers binding of a *Bacillus thuringiensis* Cry1Ab pore-forming toxin to aminopeptidase N receptor leading to insertion into membrane microdomains.. Biochim Biophys Acta.

