# Supplementary material for: Down Regulation of a Gene for Cadherin, but Not Alkaline Phosphatase, Associated with Cry1Ab Resistance in the Sugarcane Borer Diatraea saccharalis
Source: PLoS One. 2011 Oct 3;6(10):e25783. doi: 10.1371/journal.pone.0025783 (PMC3185034; doi:10.1371/journal.pone.0025783)
Supplement: Table S1 — Sequences of primers used in cDNA cloning and quantitative reverse transcriptase polymerase chain reaction (qRT-PCR) for characterization of three midgut alkaline phosphatase genes from Cry1Ab-susceptible and -resistant strains of D. saccharalis . (DOC) [file pone.0025783.s001.doc]

Table S1 Sequences of primers used in cDNA cloning and quantitative reverse transcriptase polymerase chain reaction (qRT-PCR) for characterization of three midgut alkaline phosphatase genes from Cry1Ab-susceptible and -resistant strains of *D. saccharalis*.

| **Purpose of use** | **Primer Name** | **Primer Sequence (5’-3’)** |
| --- | --- | --- |
| Specific primer for 5’RACE | DsALP1R1  DsALP1R2  DsALP2R1  DsALP2R2  DsALP3R1  DsALP3R2 | CGGGCAGCGAGTAGGATC  ACTTCTCCTGTCACACCCAAC  TTCAGCAGTGTGGGCATATG  CATACCATCACCCAAGAACATGA  GCAGCTAGTGATTTAAGCCTGG  GGTTACAGTATTAGGTCCCATACC |
| Specific primer for cDNA cloning | DsALP1F0  DsALP1R0  DsALP2F0  DsALP2R0  DsALP3F0  DsALP3R0 | TATAGATTGTCGCTCCGTCTCG  CGTTATAAAGTATTATACTTATATTTATCTAAACAAGGTACG  AGAGCAATTGACTTGCTTGTGT  GGTGGAGTGACATATATTTTGTAGTTTATTTAC  ACCGGCAGTATCAATCATGAATT CACTCGGTTTCTCTGTCTACAG |
| Specific primer for qRT-PCR | Ds18SF1  Ds18SR1  rtDsALP1F0  rtDsALP1R0  rtDsALP2F0  rtDsALP2R0  rtDsALP3F0  rtDsALP3R0 | CAAATGTCTGCCTTATCAACTTTC  GCCTTCCTTGGATGTGGTAG  GAAGAAGGGCAGTTAGGATTAAGG  TACTCAGGTGGTGATTGCATATTC  CGTTTACTCTATCGCAGCATGG  TTCAGCAGTGTGGGCATATG  CTGATTCCGCTTGTAGTGCTA  GCAGCTAGTGATTTAAGCCTGG |
| Specific primer for internal standards in qRT-PCR | rtDsALP1F1  rtDsALP1R1  rtDsALP2F1  rtDsALP2R1  rtDsALP3F1  rtDsALP3R1 | CCGTATAACACATGCGTCTCC  GCATGATCCGCAGTCACCAC  AGAGCAATTGACTTGCTTGTGT  TTGGATCGTCTCGTCCAATG  ACCGGCAGTATCAATCATGAATT  ACTGCTTGGCAAGAAACCAT |
