# Supplementary material for: Down Regulation of a Gene for Cadherin, but Not Alkaline Phosphatase, Associated with Cry1Ab Resistance in the Sugarcane Borer Diatraea saccharalis
Source: PLoS One. 2011 Oct 3;6(10):e25783. doi: 10.1371/journal.pone.0025783 (PMC3185034; doi:10.1371/journal.pone.0025783)
Supplement: Table S2 — Sequences of primers used in cDNA cloning, quantitative reverse transcriptase polymerase chain reaction (qRT-PCR), and double-stranded RNA (dsRNA) synthesis for characterization of a midgut cadherin gene from Cry1Ab-susceptible and -resistant strains of D. saccharalis . (DOC) [file pone.0025783.s002.doc]

Table S2 Sequences of primers used in cDNA cloning, quantitative reverse transcriptase polymerase chain reaction (qRT-PCR), and double-stranded RNA (dsRNA) synthesis for characterization of a midgut cadherin gene from Cry1Ab-susceptible and -resistant strains of *D. saccharalis*.

| **Purpose of use** | **Primer Name** | **Primer Sequence (5’-3’)** |
| --- | --- | --- |
| Degenerate primer* for PCR | dgDsCAD1F1  dgDsCAD1R2  dgDsCAD1F2  dgDsCAD1R3  dgDsCAD1F3  dgDsCAD1R4  dgDsCAD1F4 | ATHACNCARMGNCARGAYTAYGA  TCRTCRTTCCARTTDATNAR  YTNATHAAYTGGAAYGAYGA  GGNCCRTCDATRTCNGTNGC  GCNACNGAYATHGAYGGNCC  CCNGCNTSNARNCCRTCNTSRTC  GAYSANGAYGGNYTNSANGCNGG |
| Specific primer for 5’RACE | DsCAD1R3  DsCAD1R4 | GTGCTGATCTCTCCGTCTCT  GAATATCTCCACCCATCGAGG |
| Specific primer for cDNA cloning | DsCAD1F0  DsCAD1R1  DsCAD1F1  DsCAD1R2  DsCAD1F2  DsCAD1R0 | CTTCTTATGATAACAATCGCCTAACAAAAA  GTCTTCCAGATGTATCACCAATTGTG  TGCGTATTGACGAGGAGAGT  CATTAGTAATAGTGAAGTGTTCGATGGC  CGTGCGATGGCAAACAATC  CAGTGATATATTTTGTGCTTATAGCGC |
| Specific primer for qRT-PCR | Ds18SF1  Ds18SR1  rtDsCAD1F1  rtDsCAD1R1  DsCAD1F0  DsCAD1R4 | CAAATGTCTGCCTTATCAACTTTC  GCCTTCCTTGGATGTGGTAG  CTGGTGTCACGTTGGTAATAGTAAA  GTGCTGATCTCTCCGTCTCT  CTTCTTATGATAACAATCGCCTAACAAAAA  GAATATCTCCACCCATCGAGG |
| Specific primer for dsRNA synthesis | iDsCAD1F1  iDsCAD1R1 | TAATACGACTCACTATAGGGCTGGTGTCACGTTGGTAATAGTAAA  TAATACGACTCACTATAGGGGAATATCTCCACCCATCGAGG |

* Lepidopteran species with Genbank accession numbers of 10 cadherin sequences for degenerate primers used in PCRs are as follows: *Heliothis virescens* (AAK85198), *Helicoverpa armigera* (AAM69351), *Lymantria dispar* (AAL26896), *Manduca sexta* (AAM21151), *Bombyx mori* (BAA99406), *Pectinophora gossypiella* (AAP30715), *Ostrinia nubilalis*_A1(AAT37678), *Ostrinia nubilalis_*M1(ACK37449), *Ostrinia furnacalis* (ABL10442), *Chilo suppressalis* (ABG91735).
